# Deer Browsing Delays Succession by Altering Aboveground Vegetation and Belowground Seed Banks

**DOI:** 10.1371/journal.pone.0091155

**Published:** 2014-03-07

**Authors:** Antonio DiTommaso, Scott H. Morris, John D. Parker, Caitlin L. Cone, Anurag A. Agrawal

**Affiliations:** 1 Department of Crop and Soil Sciences, Cornell University, Ithaca, New York, United States of America; 2 Smithsonian Environmental Research Center, Edgewater, Maryland, United States of America; 3 Department of Ecology and Evolutionary Biology, Cornell University, Ithaca, New York, United States of America; University of Saskatchewan, Canada

## Abstract

Soil seed bank composition is important to the recovery of natural and semi-natural areas from disturbance and serves as a safeguard against environmental catastrophe. White-tailed deer (*Odocoileus virginianus*) populations have increased dramatically in eastern North America over the past century and can have strong impacts on aboveground vegetation, but their impacts on seed bank dynamics are less known. To document the long-term effects of deer browsing on plant successional dynamics, we studied the impacts of deer on both aboveground vegetation and seed bank composition in plant communities following agricultural abandonment. In 2005, we established six 15×15 m fenced enclosures and paired open plots in recently fallowed agricultural fields near Ithaca, NY, USA. In late October of each of six years (2005–2010), we collected soil from each plot and conducted seed germination cycles in a greenhouse to document seed bank composition. These data were compared to measurements of aboveground plant cover (2005–2008) and tree density (2005–2012). The impacts of deer browsing on aboveground vegetation were severe and immediate, resulting in significantly more bare soil, reduced plant biomass, reduced recruitment of woody species, and relatively fewer native species. These impacts persisted throughout the experiment. The impacts of browsing were even stronger on seed bank dynamics. Browsing resulted in significantly decreased overall species richness (but higher diversity), reduced seed bank abundance, relatively more short-lived species (annuals and biennials), and fewer native species. Both seed bank richness and the relative abundance of annuals/biennials were mirrored in the aboveground vegetation. Thus, deer browsing has long-term and potentially reinforcing impacts on secondary succession, slowing succession by selectively consuming native perennials and woody species and favoring the persistence of short-lived, introduced species that continually recruit from an altered seed bank.

## Introduction

Large herbivores are often considered keystone species because they can both directly and indirectly alter plant community structure and composition [1]–[3]. High densities of white-tailed deer (*Odocoileus virginianus* Boddaert) populations across North America, for example, are having strong impacts on plant communities, shifting vegetation composition and structure [4], [5]. Because deer are generalist browsers that thrive in post-agricultural habitats, they can directly impact the abundance and dispersal of seeds, and potentially alter seed bank composition during secondary succession. Deer browsing may also impact the abundance and diversity of native versus non-native plant species, as disturbance and browsing may shape community dynamics in secondary succession.

Soil seed banks play a vital role in determining vegetation structure and composition [6]–[9]. The seed bank reflects past conditions [10]–[13] and is critical for the recovery of natural areas following disturbance. For example, the seed bank is crucial for predicting patterns and trajectories in aboveground plant communities and overall ecosystem functioning [7], [9] as well as early successional dynamics following agricultural abandonment [14]–[16]. Nonetheless, relatively few studies in these systems have directly linked the composition of dormant underground propagules with aboveground vegetational dynamics.

Despite being generalists, deer are selective feeders, principally consuming woody species as well as native and non-native herbs, and can have strong indirect effects via changes in plant reproduction and competitive dynamics [1], [17]. This selectivity in diet could rapidly lead to depressed diversity of soil seed banks, which in turn is predicted to impact aboveground vegetation, especially early in secondary succession [4], [18]. A number of studies have assessed the impact of deer on aboveground plant communities, particularly in forests [1]–[3], [18]–[25], but few studies have considered how seed bank composition is affected by the presence of large herbivores such as deer. This is a critical link to understanding the long-term impacts of large herbivores as well as the regeneration potential of seed banks following disturbance such as heavy grazing [7], [26].

The primary aim of this study was to quantify the effects of deer browsing on old-field seed bank composition and aboveground dynamics during the early stages of secondary succession. In addition, we place special emphasis on the relative impacts of deer exclusion on three aspects of plants: 1) life-history (annual/biennial or perennial), 2) woody vs. non-woody species, and 3) native vs. non-native plant species. In particular, the first two groupings specifically address the impacts of deer on the early temporal dynamics of succession, as short-lived annual and biennial plants are favored early, but perennials and woody species are typically favored later in succession [27], [28]. The establishment of native vs. non-native plant species was assessed to test the hypothesis that the net impact of heavy deer browsing is to enhance the abundance and diversity of non-native plants [2]–[4], [29]. In particular, we tested the hypothesis that the net impact of heavy deer browsing is to enhance the abundance and diversity of non-native plants.

Using exclosure and deer-accessible control plots in recently abandoned old-field habitats, we predicted that under continued heavy browsing pressure, seed bank abundance and diversity would be reduced over time. Additionally, we predicted that impacts would be most severe for native herbaceous perennial species and for all woody species. We expected impacts to be mirrored between the seed bank and the composition and structure of the aboveground vegetation. Thus, we ultimately tested the prediction that the presence of deer slows plant succession and biases it towards introduced plants.

## Methods

### Study Site and Experimental Design

The property where the study occurred is owned by Cornell University and we received their permission to perform this long term study. This field study did not involve any endangered or protected species. Although the effects of browsing by wild white-tailed deer populations were monitored, there was no direct interaction or handling of deer, thus no specific animal ethics committee approval was required for this project.

In July 2005, two 15×15 m plots were each established in six blocks distributed among abandoned agricultural fields near Ithaca, NY (42°27′N, 76°26′W). Soil is poorly drained in all six blocks, which is common for this region of central New York State, and ranges from Erie Channery silt loam to Erie-Ellery Channery silt loam [30]. All blocks had historically been in agriculture but had slightly different recent histories. Blocks 1 and 2 were fallow but had not been plowed for at least 23 years prior to the start of this experiment. Block 3 was a hay field that had remained fallow and occasionally mowed for five to six years prior to start of this study. Blocks 4–6 were primarily hay fields, although corn was cultivated on each for several years prior to reverting back to a hayfield. All blocks are within 20 m of forest with the exception of block 5, which is located ∼100 m from forest. To minimize recent differences in vegetation history before initiating the experiment, we mowed existing vegetation, applied the non-selective herbicide glyphosate at the label recommended rates, and then mechanically tilled the soil using a disk harrow.

Current estimates of deer density (using motion-detection cameras) at our study site are 39 deer km^−2^ (Jay Boulanger, personal communication), which is nearly 10 times the estimated historical native density [31]. To examine the impacts of these over-abundant herbivores, each block consists of one open, deer accessible control plot and one adjacent fenced deer exclosure. Fences are 2.5 m tall with a woven high-tinsel wire, which eliminates deer access but allows for the continued free movement of other species, such as birds and small mammals. In particular, rabbits have been observed freely entering exclosure plots, thus we can attribute nearly all vertebrate browsing to deer.

### Seed Bank Sampling

Each year (2005 to 2010) soil samples were collected from each of the six blocks in late October. In both deer accessible and exclosure plots, we divided each paired 15×15 m plot into four equal subplots (NE, NW, SE, and SW directions) for ease of sampling. In each subplot, 3.5 L of soil were collected from 12 randomly spaced samples using an 8-cm diameter soil corer to a depth of 15-cm. The soil was homogenized, bagged, and stored in a cooler at 4°C before transferring to a greenhouse. Following a period of cold stratification of at least 6 weeks, each 3.5 L soil sample was mixed in a 2∶1 ratio with 2∶2∶1 mixture of peat, vermiculite, and perlite and spread to a depth of 2.5 cm in plastic flats. Any large rocks, roots, and/or rhizome pieces were removed from the soil during preparation. Each year a total of 48 flats were established, 24 flats from the deer accessible plots and 24 flats from the exclosure plots.

The flats were kept in a heated greenhouse with a day/night temperature regime of 24/21°C and a 14-h photoperiod. Flats were watered as required and fertilized once weekly with 200 ml of a 21-5-20 N-P-K solution. Over a period of seven weeks the flats were regularly inspected to determine the number of emerged seedlings. Seedlings were identified to species, counted, and removed as soon as accurate identification was possible. Following this period, flats were transferred back to the cooler for an additional three weeks of cold stratification to stimulate additional germination and emergence of seeds that had not emerged during the first germination sequence. Flats were then returned to the greenhouse under the same conditions as the first germination cycle; however, the soil was stirred in each flat to redistribute seeds within the soil profile. Seedling emergence was recorded for an additional seven weeks. Seedling emergence totals for a given year are based on the combined emergence from the two germination cycles.

### Aboveground Vegetation Sampling

In fall of 2005, two months after plowing and imposing the deer exclusion treatment, we sub-sampled aboveground biomass and estimated percentage bare soil in all subplots. We clipped all vegetation from one 0.25 m^2^ quadrat in each subplot, sorted to species, and dried to constant temperature at 60°C. Percentage bare soil in each 0.25 m^2^ quadrat was estimated visually. Then, in each year from 2006–2008, we recorded the percentage cover of herbaceous and woody species, resulting in a total of four vegetation censuses: June 2006, September 2006, August 2007, and July 2008. In 2006, cover was estimated by recording each species that intersected 20 randomly selected locations along each of 10 randomly placed transect tapes bisecting each plot. At each location we also recorded the maximum vegetation height and whether the plant was flowering or not. Cover for each species was then calculated as the percent of locations occupied by that species. In years 2007 and 2008 cover was visually estimated as percentage by species in each subplot to the nearest 1% by three independent observers, taking the average of all three estimates and then averaging over the entire plot. In all three years total cover was allowed to exceed 100% to account for overlapping vegetation. Total species richness was assessed in the entirety of each plot, with a 0.1% cover given to species present in only trace amounts. Woody stems in each plot were counted in years 2005–2009 and 2012 to assess the long-term implications of deer browsing on forest regeneration following agricultural abandonment.

### Data Analyses

For aboveground data collected in 2005, the first year of abandonment, we used separate linear mixed effects models (R version 2.14.2, R Foundation for Statistical Computing; package *lme4* [32] to assess whether percentage bare soil and aboveground biomass were impacted by deer browsing. Plot (n  = 12) was the unit of replication, and residuals were checked for normality and equal variance. Deer exclusion treatment was a fixed factor and block was a random factor. For seed bank data collected in 2005–2010, we also used linear mixed effects models to assess whether the following variables differed by browsing treatment: germinant species richness, species diversity, total germinant abundance, the proportional abundance of annual/biennial plants, and the proportional richness and abundance of native species. We conducted similar analyses for aboveground vegetation data collected in 2006–2008, and for woody stem density (2005–2009, 2012). In all models deer exclusion treatment and time were fixed factors; block was a random factor, and plot was the unit of replication. For all models we compared the significance of the treatments by comparing the likelihood of models with and without each predictor. Species diversity was calculated using the Shannon-Wiener Index (H′) calculated as follows: H = −Σ(Pi ln[Pi]), Where, Pi is the relative abundance of the number of individuals in species i, over the total number of individuals of all species [33], [34]. This results in a diversity value ranging from 0, indicating low community diversity, to 4, indicating high diversity.

To assess the extent of browsing and whether this influenced plant height and reproduction, we used binomial logistic regression to determine the odds of a plant being browsed in our treatments in 2006 (the only year we collected height and browsing frequency data). The binary matrix included the number of browsed versus unbrowsed plants in each plot, with plot, transect, and species included as random effects. We then tested whether plants in open plots were shorter than caged plants, where we first aggregated plant height by species in each plot. We then used block and species as random effects in a mixed effects model. Height was log transformed to achieve normality. A majority of observed browsing occurred on just three species: *Erigeron annuus* (native annual), *Rumex crispus* (introduced perennial), and *Silene alba* (introduced biennial). For these three species we tested whether plants in open plots were shorter and less likely to be flowering than plants in caged plots. For the latter we used binomial logistic regression with block, transect, and species included as random effects.

To address similarities between aboveground vegetation and the seed bank, we used analyses of covariance (ANCOVAs) to assess whether the richness, diversity, and structure of aboveground vegetation could be predicted by what we found in the seed bank. This tested, for example, whether the species richness of aboveground vegetation in 2006 was a function of the species richness of plants available in the soil seed bank in 2005. In these analyses, plots were the unit of replication, and deer and time were fixed factors, and seed bank metrics were the covariates.

## Results

**Figure 1:**
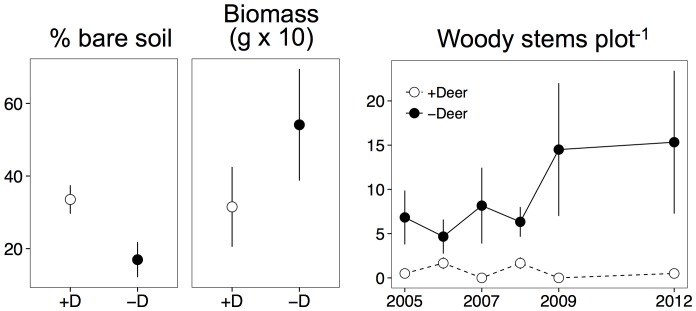
Impacts of deer browsing on initial establishment of the plots (2 months after imposition of the treatment). Shown are means ± SE for percentage bare soil, the aboveground dry biomass of subsamples (0.25 m^2^), and the number of woody plants recruited into the plots. All responses were statistically significant (P≤0.05).

With the sole exception of *Asclepias syriaca*, all of the vegetation that emerged following plowing was from the seed bank (personal observations). After two months of initiating the experiment, the percentage bare soil and aboveground biomass were both impacted by deer exclusion (percent bare soil: χ^2^ = 6.441, P  = 0.011; aboveground biomass: χ^2^ = 3.998, P  = 0.046). In particular, deer browsing nearly doubled the amount of bare soil but reduced aboveground biomass by ∼43% (Fig. 1).

**Figure 2:**
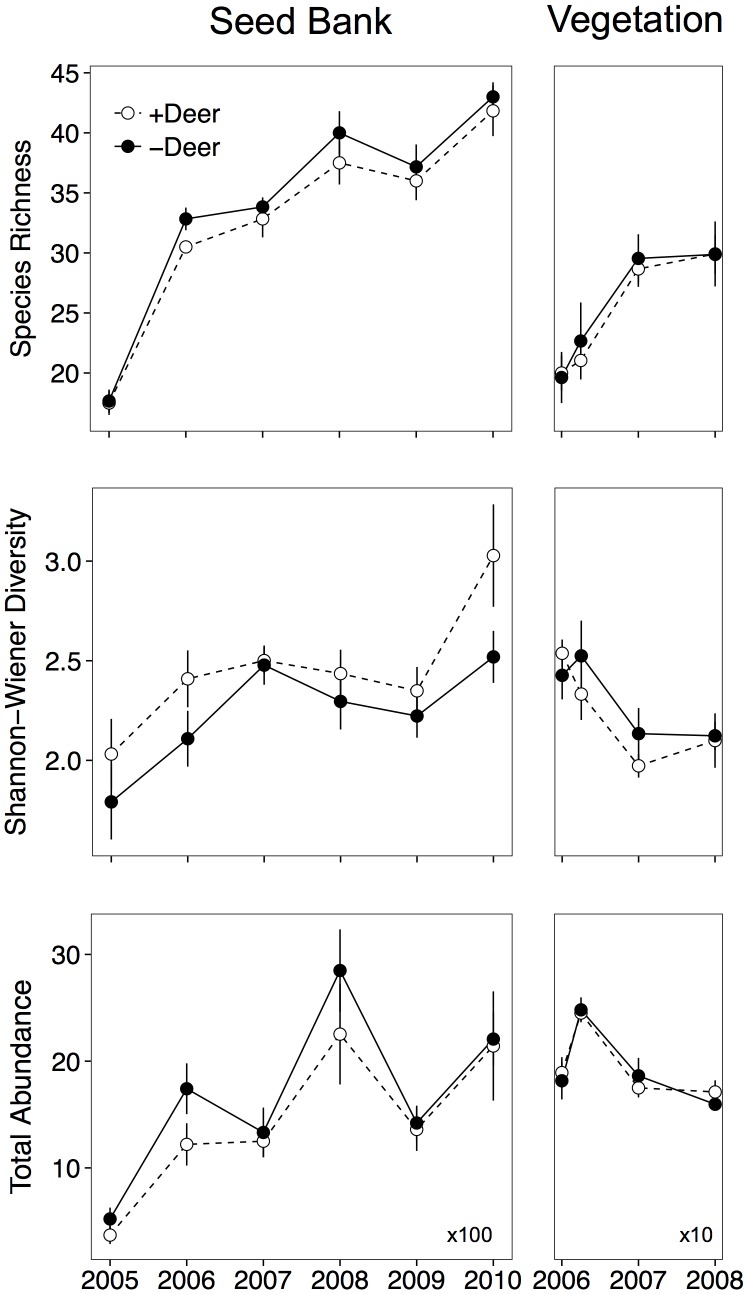
Impacts of deer browsing on species richness, diversity, and total abundance of germinants from the soil seed bank (left panels) between 2005 and 2010, and aboveground vegetation (right panels) in years 2006–2008. (Shown are means ± SE).

**Figure 3:**
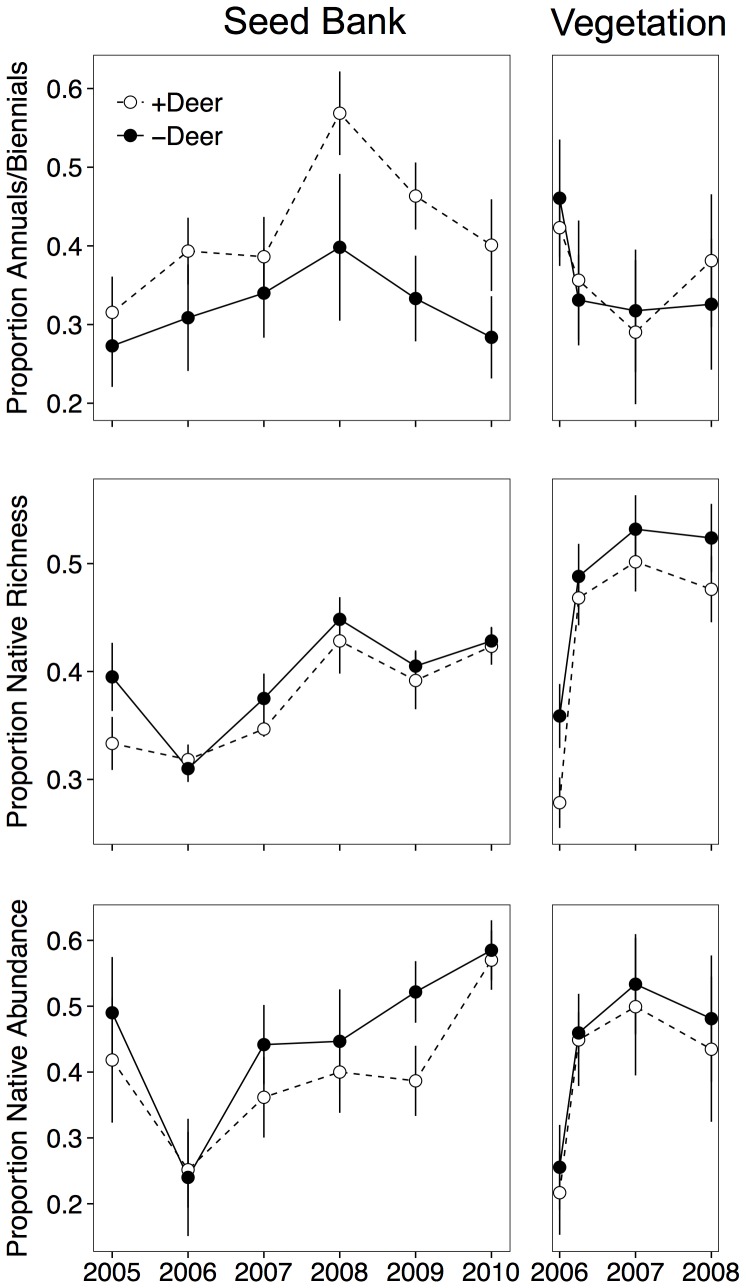
Impacts of deer browsing on the relative abundance of annuals/biennials, the proportion of species that are native, and the proportion of total germinants/cover that was native in the soil seed bank (left panels) and aboveground vegetation (right panels). Shown are means ± SE.

A total of 110,885 seedlings from 105 species emerged from the seed bank over the six sampling seasons across all plots. The species richness, diversity, and total abundance of germinants increased over time and were all affected by deer (Fig. 2; Table 1). Deer browsing in open areas resulted in a significant 4% decrease in germinant species richness, a 10% increase in diversity, and a 15% increase in total germinants (Fig. 2; Table 1). Thus, although more seedlings emerged from exclosure plots and these plots had modestly higher species richness, the deer accessible plots were more diverse. Deer also altered the general structure of the plant community, causing a 31% increase in short-lived monocarpic plants in the seed bank, a 5% decrease in the number of native relative to introduced species, and a 12% decrease in the abundance of native species in the seed bank (Fig. 3; Table 1). Very few woody plants emerged from the soil seed bank (10 individuals in total).

**Table 1:** Results of linear mixed models testing effects of deer exclosure and year on measures of plant community structure from the soil seed bank and aboveground vegetation.

Response	Deer exclusion	P	Year	P
**Seed Bank Germinants**				
Seedling richness	3.706	**0.054**	143.58	**<0.001**
Seedling diversity (Shannon-Wiener index)	10.244	**0.001**	42.647	**<0.001**
Total germinants	5.234	**0.022**	81.972	**<0.001**
Relative abundance annuals and biennials	17.863	**<0.001**	23.988	**0.002**
Proportion native species germinated	4.679	**0.031**	55.309	**<0.001**
Proportion native germinants	4.784	**0.029**	42.948	**<0.001**
**Aboveground Vegetation**				
Aboveground species richness	0.323	0.570	48.922	**<0.001**
Aboveground diversity (Shannon-Wiener index)	1.088	0.297	25.617	**<0.001**
Aboveground cover	0.027	0.870	40.953	**<0.001**
Relative cover of annuals and biennials	0.013	0.911	7.258	0.064
Woody stems	17.731	**0.035**	3.678	0.597
Proportion native species	11.046	**<0.001**	65.777	**<0.001**
Proportion native cover	0.874	0.350	27.216	**<0.001**

The year-by-deer exclusion interaction was never significant and subsequently dropped from all models; df  = 5 for germinant models and df  = 3 for aboveground vegetation models. Initial values shown are **χ** ^2^ values for likelihood tests excluding treatment of interest. Bold values denote statistical significance at P≤0.05.

A total of 142 species were recorded in cover surveys across all plots from 2006 to 2008. In contrast to results from the seed bank, the presence of deer did not affect aboveground species richness (P  = 0.570), diversity (P  = 0.297), total cover (P  = 0.870), or the relative cover of annuals versus perennials (P  = 0.911). Most of these variables, however, varied with time (year effect was P≤0.001 for all variables except annuals/perennials, which was P  = 0.064). However, deer reduced the number of woody stems (P  = 0.035) and strongly reduced the proportion of native relative to introduced species (P<0.001) in open plots (Figs. 1 & 3, Table 1). Five of the six exclosure plots were colonized by woody species which had established by seed and ranged in size from 0.5 to 1 m tall (Fig. 1). These species included *Rhus typhina* L. (staghorn sumac), *Populus deltoides* Marshall (common cottonwood), *Acer negundo* L. (boxelder), and *Robinia pseudoacacia* L. (black locust).

Browsing pressure in the first full year was selective but appears to have had broad impacts on plant community structure. Overall, the odds of being browsed were 95% lower for plants in the caged versus the open plots (χ^2^ = 235.1, P<0.001), plants in caged plots were 30% taller (38.4±2.1 cm in open versus 29.6±1.6 cm in caged plots, χ^2^ = 13.3, P<0.001), both indicating that the presence of rabbits in caged and uncaged plots likely had minimal impacts. Although casual observations indicated browsing on numerous species, over 66% of observed deer browsing in our quantitative transect surveys in 2006 occurred on just three species: *Erigeron annuus* (a native annual), *Rumex crispus* (an introduced perennial), *Silene alba* (an introduced biennial), with no other herbaceous species incurring greater than 6% of the observed browsing pressure (exclusive of woody species that were generally not captured in this survey). *S. alba* was one of the most abundant species, with upwards of 38% cover. Greater than 37% of *S. alba* plants were browsed in the open plots (versus ∼1% in the deer exclusion plots, χ^2^ = 116.7, P<0.001), resulting in plants that were 30% shorter (48.7 cm ±1.4 cm in deer plots, 69.3±1.7 cm in deer exclusion plots, χ^2^ = 53.02, P<0.001) and flowered 38% less frequently (60.3% ±8.8% of plants flowered in deer plots, 96.9% ±2.5% of plants flowered in deer exclusion plots, χ^2^ = 67.7, P<0.001). Similarly, *E. annuus* was 49% shorter in open plots (52.2±3.6 cm in deer plots, 101.1 cm ±2.7 cm in deer exclusion plots, χ^2^ = 58.6, P<0.001) and flowered 35% less frequently (37.5% ±9.6% of plants flowered in deer plots, 58.1% ±23.7% of plants flowered in deer exclusion plots χ^2^ = 34.4, P<0.001). *R. crispus* was 33% shorter in open plots (58.4±2.7 cm in deer plots, 87.1 cm ±2.7 cm in deer exclusion plots, χ^2^ = 58.6, P<0.001) and flowered 29% less frequently (63.1% ±5.2% of plants flowered in deer plots, 89.1% ±5.0% of plants flowered in deer exclusion plots, χ^2^ = 71.6, P<0.001). Similarly, the abundance of *R. crispus* was also suppressed by deer in the seed bank (mean ± SE germinants per year 2005–2010: 48.14±12.66 in deer plots, 154.19±35.14 in deer exclusion plots, P<0.001), and in the aboveground surveys (mean ± SE cover 2006–2008: 9.5±2.4 in deer plots, 19.5±5.3 in deer exclusion plots, P  = 0.042).

### Correspondence between Aboveground Vegetation and Seed Bank Diversity and Composition

**Figure 4:**
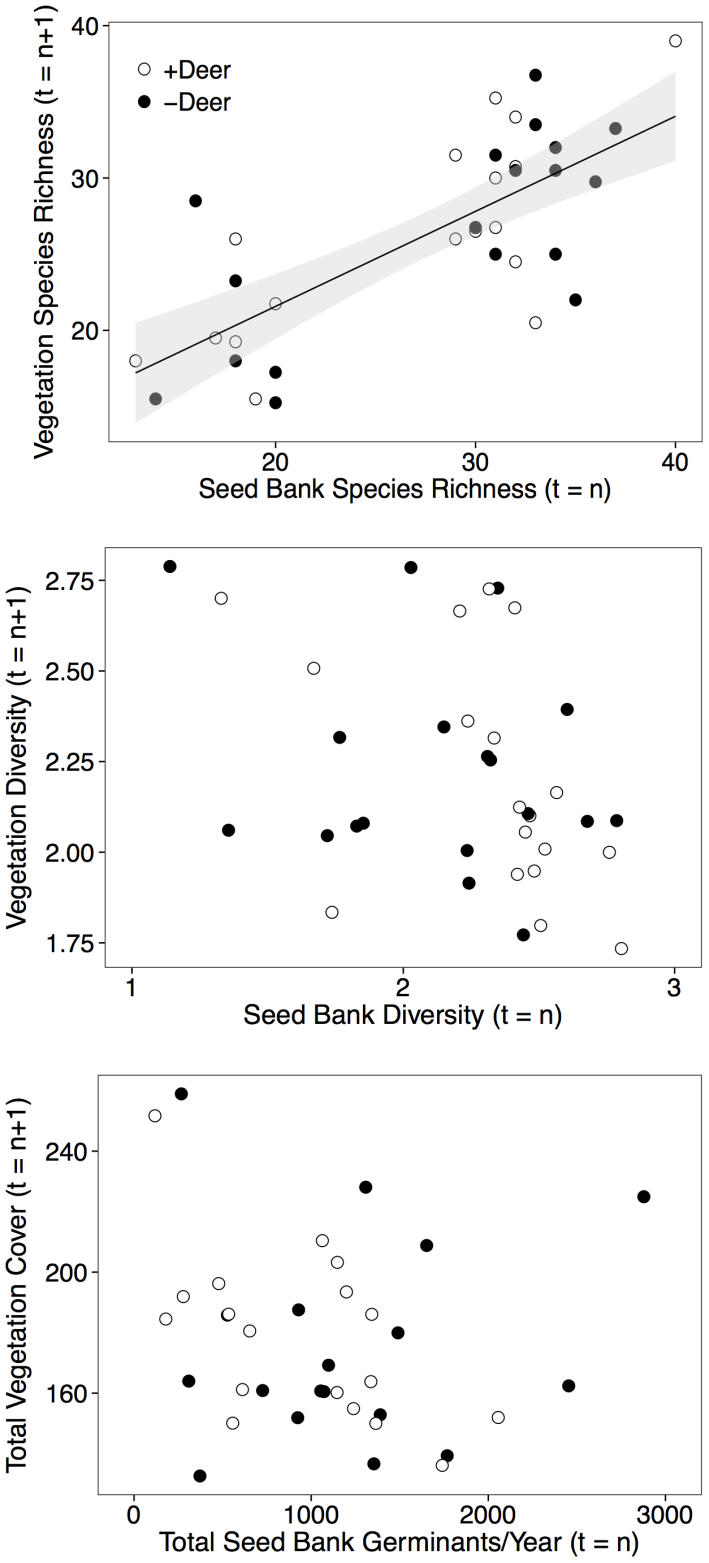
Across year correlations for species richness, diversity, and total abundance in the seed bank versus aboveground vegetation. Statistically significant best fit lines are shown. Statistics are given in Table 2.

**Figure 5:**
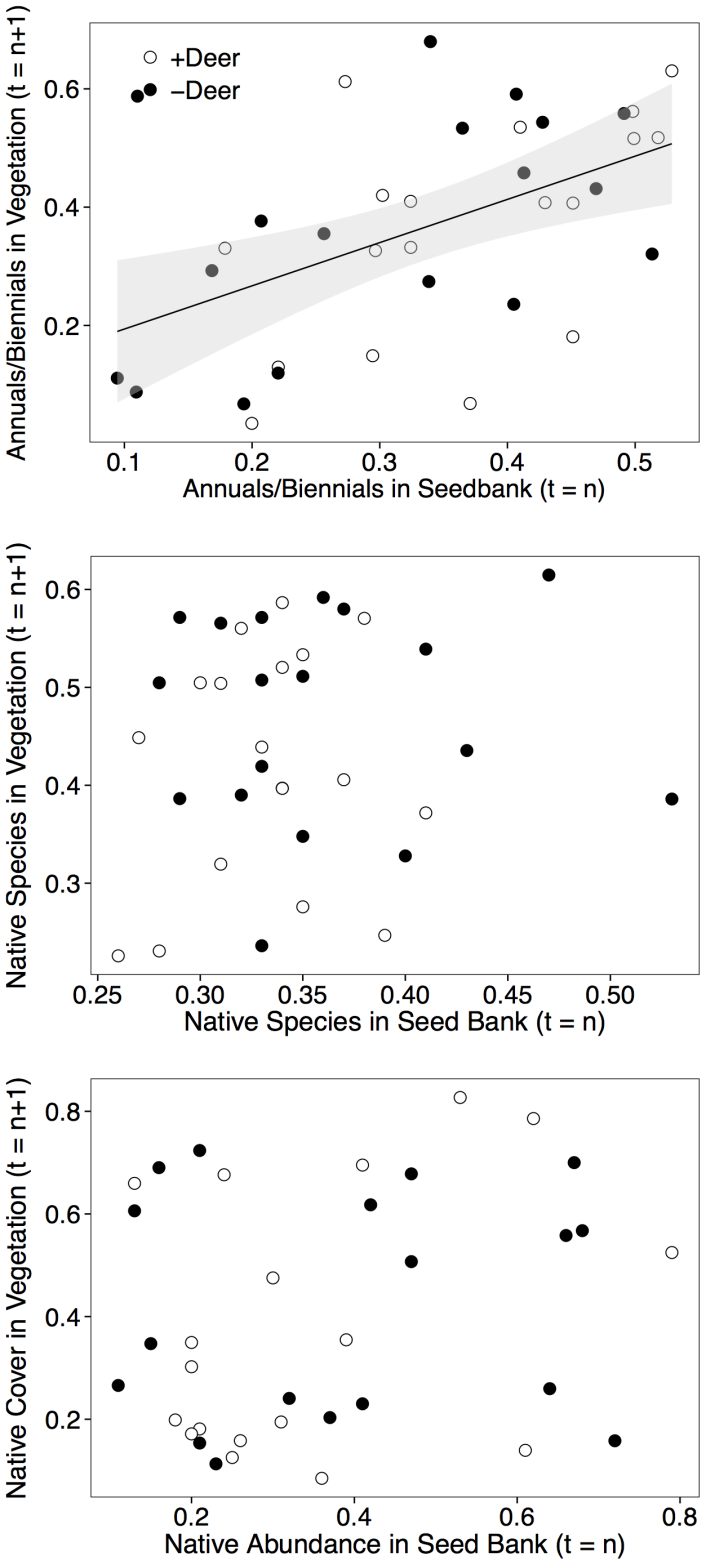
Across year correlations for relative abundance of annuals/biennials, native species, and native abundance in the seed bank versus aboveground vegetation. Best fit lines are shown when significant. Statistics are given in Table 2.

Deer had strong impacts on every aspect of the seed bank species’ structure that we measured (Figs. 2 & 3), but we were only able to detect a relationship among seed bank and vegetation structure for two variables: species richness and the relative abundance of short-lived species. For example, ANCOVAs showed that there was a positive relationship between the species richness of the seed bank and the species richness of the aboveground vegetation, but there was no effect of deer exclusion on this relationship (Fig. 4, Table 2). There was neither a correlation between aboveground diversity and seed bank diversity, nor between aboveground total cover and the total abundance of seed bank germinants (Fig. 4, Table 2). Similar to the result for species richness, the relative abundance of short-lived species in the aboveground vegetation was positively related to their relative abundance in the seed bank (Fig. 5, Table 2). The relative abundance of native species in the aboveground vegetation, however, was not related to their relative abundance in the seed bank.

**Table 2:** Results of ANCOVAs testing effects of year, deer exclosure and the previous year’s seed bank structure on measures of aboveground plant community structure.

Aboveground Response	Year	Deer Exclusion	Seed Bank Covariate	Deer*Covariate
Species Richness	t = 0.199; P = 0.843	t = 0.224; P = 0.825	t = 2.595; **P = 0.014**	t = −0.325; P = 0.747
Diversity	t = −2.332; **P = 0.026**	t = −1.155; P = 0.257	t = −1.261; P = 0.217	t = 1.191; P = 0.243
Abundance	t = −0.905; P = 0.373	t = −1.576; P = 0.125	t = −1.367; P = 0.181	t = 1.692; P = 0.101
Annuals/Biennials	t = −2.308; **P = 0.028**	t = 0.953; P = 0.348	t = 3.082; **P = 0.004**	t = −0.647; P = 0.522
Relative Native Species Richness	t = 4.925; **P<0.001**	t = 0.316; P = 0.754	t = 0.321; P = 0.750	t = −0.091; P = 0.928
Relative Native Abundance	t = 2.715; **P = 0.011**	t = 1.111; P = 0.275	t = 1.883; P = 0.069	t = −1.071; P = 0.293

Bold values denote statistical significance at P≤0.05.

## Discussion

The impacts of deer browsing on composition and diversity of seed banks and above ground vegetation in old-field plots in this 6-year study occurred relatively early and have persisted. The effects of very high deer density populations (≈39 deer km^−2^) on plant composition are most striking in terms of impacts on plant life-history (e.g. annual/biennial vs perennial), the nearly complete suppression of woody plants, and the abundance of introduced species. Effects of deer were generally stronger for the seed bank relative to the aboveground vegetation, but there were similarities in the species richness and relative abundance of short-lived species in the seed bank and aboveground vegetation.

While the effects of selective deer browsing on plant community composition and diversity and in delaying forest succession in North America have been well documented (e.g. [3], [4], [35]–[37]), less is known about how deer browsing may affect these parameters at earlier secondary successional stages such as in recently established old-field communities (e.g. [38], [39]). In general, species diversity and overall species richness tend to decrease as secondary succession proceeds [6], [8]. We found that in the absence of deer, the species richness of the seed bank increased as did the total number of emerged seedlings, but that species diversity declined relative to deer-accessible plots. A likely reason for these findings is that deer browsing severely reduced growth and reproduction of the highly palatable perennial herb, *Rumex crispus,* thus limiting its early dominance in deer accessible plots.

The finding of higher species diversity in plots accessible to deer supports previous research showing that selective deer browsing delays or alters successional trajectories [35], [36]. In contrast, Bowers [38] reported lower plant species diversity in a 4-yr old-field community in Virginia, USA that experienced either high or low intensities of herbivory from different mammalian herbivores, including deer, relative to intermediate levels of herbivory. The major effect of herbivory was to alter competitive hierarchies in the plant community rather than directly causing plant death. Cadenasso et al. [39] found that mammalian herbivore exclusion (i.e., deer, cottontail rabbits, and meadow voles) in a 1-yr and 17-yr old field community in New Jersey, USA influenced primarily the structure (i.e., height) of the plant community rather than its composition.

In deer accessible plots, we found significant decreases in the proportion of native species emerging from the seed bank relative to deer exclusion plots. Native species that were most impacted by deer browsing pressure included *Solidago altissima*, *Oxalis stricta*, and *Populus deltoides*. There is still debate as to whether generalist native browsers such as deer have a greater impact on native or non-native species [40], [41]. The presence of deer favored the persistence of short-lived species such as annuals and biennials (e.g. *Chenopodium album*, *Ambrosia artemisiifolia*, *Barbarea vulgaris*), thus altering secondary successional trajectories. After six years, perennials comprised 66% of emerged seedlings from the deer exclosure plots and 52% of seedlings from the deer accessible plots. These differences were apparent for the seed bank as early as the end of the first growing season after the establishment of exclosures (Figs. 1–3). It is unclear whether the impacts of deer browsing on short-lived non-native species will have long-term impacts on the community.

In the absence of deer browsing (i.e., exclosure plots), succession appears to be progressing at a faster rate than in plots that were accessible to deer. This trend is consistent with our findings of the relative abundance of annuals, biennials, and perennials including woody species over time (Figs. 1–3). Cadenasso et al. [39] also reported significant growth reductions from herbivores, including deer, of palatable woody species such as *Acer rubrum* and *Cornus florida* relative to growth in exclosures. Our data support their general conclusion that herbivorous mammals play a critical role in old-field succession, especially in the important shift of dominance from herbaceous to woody vegetation.

Deer nearly eliminated woody succession in our plots, consistent with several studies showing that woody species can suffer severe damage from deer browsing [17], [42]. Only two of the deer accessible plots contained woody species that emerged from the soil seed bank (*Rhus typhina* and *Robinia pseudoacacia*). Nonetheless, aboveground, we found substantial numbers of these species (as well as cottonwoods and boxelders) in the protected plots, with some evidence of individuals being browsed in open access plots. Glenn-Lewin et al. [43] suggest that disturbance and successional transition are intimately linked. In the deer accessible plots, few or no woody species were recorded in 2006, 2007, or 2008, which indicates that the small saplings had not grown tall enough to escape browsing, which likely severely impaired their growth and survival [1], [39], [44], [45], which ultimately will impact successional trends.

We only found correspondence between the seed bank and aboveground vegetation for species richness and the abundance of short-lived species. This is consistent with studies showing that in agricultural cropping systems that experience frequent disturbance, or, in recently abandoned agricultural fields, the species composition of the seed bank is similar to that of the aboveground vegetation [6], [7], [46]. The frequent disturbance in these systems favors the establishment and dominance of annual species with high reproductive outputs. For example, in deer accessible plots which suffer from regular disturbance from deer, 77% of seedlings emerging from the seed bank were also found in the aboveground vegetation surveys, while 47% of species sampled in the aboveground surveys were also recorded from the seed bank samples. In the exclosure plots, 74% of the species that emerged from the seed bank were found in the aboveground vegetation surveys, and 49% of species recorded in the aboveground vegetation were found in the seed bank. It is possible that the sampling time frame was not long enough for differences in seed bank composition to be observable following cessation of browsing in exclosure plots. In general, the similarity in species composition between the seed bank and aboveground vegetation declines when the frequency or intensity of disturbance decreases over time as succession proceeds [7], [11], [13], [47]. A possible explanation for this increasing dissimilarity is that seed banks establish over long periods of time from vegetation that may no longer be present in a given habitat [6], [7].

In deer-accessible plots, 23% of species that emerged as seedlings were found only in the seed bank due to factors such as seed longevity, dormancy, shape, size, dispersal mode, and location in the soil profile [48], [49]. Most of these species were long-lived or dispersed by wind (*Atriplex patula* L. (spreading atriplex), *Polygonum convolvulus* L. (wild buckwheat), *Chenopodium glaucum* L. (oakleaf goosefoot), *Epilobium ciliatum* Raf. (fringed willowherb) and *Erigeron philadelphicus* L. (Philadelphia fleabane)). As in the deer accessible plots, species in exclosure plots found only in the seed bank were largely wind dispersed or long-lived. Due to higher species density and successional progression in the exclosure plots, a thicker layer of litter may be acting as a physical barrier preventing seed germination and emergence as well as seed recruitment into the seed bank [50]. In deer accessible plots, seeds of species found only in the above-ground vegetation −53% of species sampled - may have been unable to reach the soil surface due to predation and size, as deer are known to consume the seeds of many different plant species, particularly those of large-seeded species (such as *Rumex crispus*) [29], [51]. While the seed bank was dominated by herbaceous species, the aboveground vegetation in the exclosure plots contained numerous woody species (*Acer negundo*, *Rhus typhina*, *Robinia pseudoacacia*, and *Carya alba* L. (mockernut hickory)). In general, these woody species have rather transient seed banks and thus these findings are not unexpected. Therefore, as succession progresses, we expect that trajectories between the two deer access treatments will continue to diverge. Lastly, these findings suggest that deer activity is rapidly influencing seed bank composition in old field systems, most likely through selective browsing of the aboveground vegetation, and will have long-term impacts on secondary succession following agricultural abandonment.

## Conclusions

The use of deer accessible and exclosure plots serves as a model to assess the direct and indirect impacts deer pose to plant communities. Our findings to date indicate that deer browsing plays a major role in determining the diversity, abundance, and successional progression of plant species in both the seed bank and the aboveground vegetation, likely putting deer accessible and non-accessible areas on divergent trajectories. As this study progresses, we expect that the plant communities in the six paired plots will continue to diverge as the seed bank and the aboveground vegetation continue to be affected by heavy browsing pressure thereby altering community development dynamics. Ultimately, although deer favor short-lived non-native species, this effect will likely be overshadowed by native perennials. In contrast, the seed bank and aboveground vegetation in the exclosure plots will likely continue to shift towards dominance by shade tolerant herbaceous perennials and woody species, a successional pathway common to the region. Thus, deer browsing has long-term and potentially reinforcing impacts on secondary succession, slowing succession by selectively consuming native perennials and woody species, and favoring the persistence of short-lived, introduced species that continually recruit from an altered seed bank.
